# USP14 inhibits mitophagy and promotes tumorigenesis and chemosensitivity through deubiquitinating BAG4 in microsatellite instability-high colorectal cancer

**DOI:** 10.1186/s10020-025-01182-w

**Published:** 2025-05-02

**Authors:** Zhiyong Wang, Cheng Yu, Gengchen Xie, Kaixiong Tao, Zhijie Yin, Qing Lv

**Affiliations:** Department of Gastrointestinal Surgery, Union Hospital, Tongji Medical College, Huazhong University of Science and Technology, Wuhan, 430022 China

**Keywords:** Colorectal cancer, USP14, Mitophagy, Tumorigenesis, Oxaliplatin

## Abstract

**Background:**

Mitophagy, essential for cellular homeostasis, is involved in eliminating damaged mitochondria and is associated with cancer progression and chemoresistance. The specific impact of mitophagy on microsatellite instability-high (MSI-H) colorectal cancer (CRC) is still under investigation. Ubiquitination, a post-translational modification, is essential for controlling protein stability, localization, and function. This study identifies USP14, a deubiquitinating enzyme, as a key regulator of mitophagy in MSI-H CRC.

**Methods:**

A deubiquitinating enzyme (DUBs) siRNA library screening identified USP14 as a key regulator of mitophagy. Tissue samples from patients were analyzed using immunohistochemistry and Western blot. USP14 knockdown cell lines were generated using lentiviral transfection. Protein interactions between USP14 and BAG4 were confirmed by co-immunoprecipitation, while quantitative PCR was used to measure gene expression. Mitochondrial proteins were extracted to analyze mitophagy, and flow cytometry was used to assess apoptosis. Finally, a mouse xenograft model was employed to study USP14’s role in tumor growth and oxaliplatin sensitivity.

**Results:**

Screening reveals that USP14 inhibits mitophagy and CRC (MSI-H) show high USP14 expression which correlates with poor prognosis. Functional analyses reveal that knocking down USP14 reduces tumor growth, and increases sensitivity to oxaliplatin. Mechanically, USP14 inhibits mitophagy by K48-deubiquitinating and stabilizing BAG4 at K403, which prevents the recruitment of Parkin to damaged mitochondria. The significant clinical relevance of USP14, BAG4, and PRKN are proved in tumor tissues.

**Conclusions:**

The study highlights the USP14/BAG4/PRKN axis as a critical pathway in CRC (MSI-H), suggesting that targeting USP14 could inhibit tumor progression and improve chemotherapeutic outcomes. These findings underscore the importance of ubiquitination and mitophagy in cancer biology, indicating a potential therapeutic target for MSI-H CRC.

**Supplementary Information:**

The online version contains supplementary material available at 10.1186/s10020-025-01182-w.

## Background

Colorectal cancer (CRC) ranks among the most prevalent cancers worldwide, with rising incidence and mortality rates each year (Ahnen et al. 2014). Globally, CRC accounts for over 1.93 million new cases and more than 953,000 deaths annually (Sung et al. 2021). One well-described genetic subset of CRC is tumors with mismatch-repair deficiency (dMMR), which are found in 15% of all patients with CRC, resulting in a very high tumor mutation burden as well as altered microsatellite sequences that render these tumors high in microsatellite instability (MSI-H). Currently, the treatment for CRC mainly includes surgery, targeted therapy, radiotherapy, and chemotherapy (Pan et al. 2023). Generally, chemotherapy is the most classic and commonly used treatment method for advanced CRC and can be used at different stages of treatment, usually as an adjunct therapy for patients with advanced CRC after surgery. Currently, the chemotherapy regimen containing oxaliplatin is the most commonly used for patients with advanced CRC, and chemotherapy resistance is the main reason for treatment failure (Zhang et al. 2022a). Therefore, it is necessary to identify effective therapeutic targets to improve chemotherapy sensitivity and thereby improve the survival and prognosis of CRC (MSI-H) patients.

Mitophagy is an autophagic process dedicated to eliminating damaged and dysfunctional mitochondria in cells (Wang et al. 2023). This process is crucial for maintaining the stability of the cellular environment and cellular functions. In response to different stimuli, mitochondria experience depolarization, leading to the recognition of damaged organelles by autophagosomes (Jang et al. 2021). These autophagosomes then merge with lysosomes to finalize the degradation process. It helps prevent cell stress and apoptosis caused by damaged mitochondria, thus protecting cells from harm. Mitophagy plays a crucial role in diverse physiological and pathological contexts, including cellular aging, neurodegenerative disorders, cardiovascular diseases, and cancer (Onishi et al. 2021). Mitophagy can reduce the cellular stress response caused by mitochondrial dysfunction, and by maintaining the integrity of mitochondrial function, cancer cells become more aggressive and tolerant to chemotherapy drugs. In CRC, mettl14-dependent pri-miR-17 maturation regulation of mitochondrial homeostasis induces chemotherapy resistance in CRC (Sun et al. 2023). Mitophagy contributes to the resistance of human CRC stem cells (Yan et al. 2017). In fact, researchers are exploring how to slow tumor growth and overcome chemotherapy resistance by regulating mitophagy, but the upstream and downstream regulatory mechanisms are currently unclear.

Deubiquitinating enzymes (DUBs) are a specific class of enzymes whose main function is to remove ubiquitin tags from proteins (Collins and Goldberg 2017). Ubiquitin is a small protein molecule that regulates the stability, location, and activity of proteins through ubiquitination. Deubiquitinating enzymes can regulate mitophagy in various ways (Tsefou and Ketteler 2022). They can remove ubiquitin tags from the surface of damaged mitochondria, thereby regulating the occurrence and progression of mitophagy. Deubiquitinating enzymes participate in mitochondrial quality control by affecting the activity of key regulatory factors of mitophagy. For example, deubiquitinating enzymes can affect the ubiquitination state of PINK1, thereby affecting its regulatory role in mitophagy (Pirooznia et al. 2022). Additionally, deubiquitinating enzymes can directly or indirectly participate in the signaling pathways of mitophagy, as well as the function and interactions of PINK1-Parkin or other proteins involved in mitophagy (Yi et al. 2024). In this study, using a deubiquitinating enzyme library screening, we found that USP14 is a key regulatory molecule of mitophagy in CRC (MSI-H). USP14 can inhibit mitophagy and promote tumorigenesis and resistance to oxaliplatin. USP14 stabilizes BAG4 by deubiquitination, which subsequently inhibits Parkin-mediated mitophagy, promotes tumorigenesis, and decreases the sensitivity of CRC (MSI-H) cells to oxaliplatin chemotherapy. High USP14 expression correlates with poor prognosis in CRC (MSI-H) patients. This study aims to investigate the role of the USP14/BAG4/PRKN axis in CRC (MSI-H) progression and its impact on chemotherapy sensitivity. We hypothesize that USP14 regulates tumor growth and response to chemotherapy through interactions with BAG4 and PRKN, thereby influencing mitophagy and apoptosis pathways.

## Materials and methods

### Cell culture and reagents

This study utilized human MSI-H CRC cell lines LoVo and SW48, sourced from the American Type Culture Collection (ATCC) and maintained according to established protocols (Zheng et al. 2022). All procedures were conducted under sterile conditions, and the incubator was set at 37 °C with 5% CO_2_. LoVo and SW48 cells were subcultured every 3 days, using 0.25% trypsin–EDTA (Gibco) for 2 min to dissociate them. The enzyme reaction was halted, and the resulting cell suspension was transferred to DMEM containing 10% FBS. Cells were collected by centrifugation (800 rpm, 2 min) and resuspended before being plated into suitable culture containers for further experimentation. Routine PCR and immunofluorescence were employed to monitor mycoplasma contamination, and the cell line integrity was confirmed through STR analysis. Oxaliplatin (S1224), USP14 inhibitor (IU1, S7134), Cycloheximide (S7418) and MG132 (S2619) were purchased from Selleck Chemicals (USA).

### DUBs library screen

The human deubiquitylases were screened using the Dharmacon siGENOME SMARTpool siRNA library for human DUBs (Mikl et al. 2022). Briefly, LoVo cells were added to the rehydrated Dharmacon RTF siRNA library plates. Cell lysates were extracted after 72 h, and TOM22 expression, a mitochondrial marker protein, was quantified using Western Blotting. We mainly focused on the 41 DUBs filtered from The Cancer Genome Atlas (TCGA) database which significantly (*p* < 0.001) expressed differently between CRC and normal tissues (Sakita et al. 2022).

### Tissues collection

The study utilized 40 human CRC tissues (MSI-H) and adjacent controls from patients who consented and underwent surgical resection at Wuhan Union Hospital. Fresh tissues were collected by the attending physician under sterile conditions during surgery. Each sample was promptly rinsed with cold phosphate-buffered saline (PBS) to eliminate blood and impurities, then either fixed in 4% formaldehyde or quickly frozen in liquid nitrogen for storage at − 80 °C for subsequent analysis.

### Plasmids and transfection

Plasmids were sourced from Genecreate Biotechnology (Wuhan, China) and extracted from Escherichia coli using the Qiagen Plasmid Mini Kit (Germany). DNA concentration and purity were evaluated with a NanoDrop spectrophotometer, yielding an A260/A280 ratio between 1.8 and 2.0. Transfections were conducted according to the Lipofectamine 2000 protocol (Invitrogen, USA). Human CRC cells were cultured in DMEM supplemented with 10% fetal bovine serum and seeded into 6-well plates to achieve approximately 70% confluence 1 day before transfection. For each well, 2 µg of plasmid DNA was mixed with 5 µL of Lipofectamine 2000 and incubated with serum-free Opti-MEM (Invitrogen) for 20 min before being applied to the cells. Cells were cultured for 48 h prior to collection for analysis.

### USP14 knockdown cell lines

USP14 knockdown cell lines were generated via a lentiviral system. Lentiviral particles were generated in HEK293T cells utilizing a Virus Packaging Kit from Genomeditech, China. SW48 and LoVo cells underwent infection followed by a 2-week selection period using 2 µg/mL puromycin. Two distinct shRNA sequences were used to produce lentiviral RNA targeting USP14: shUSP14#1: 5′-GCAGCCAAATACAAGTGACAA-3′, shUSP14#2: 5′-CCCAAGATTCAGCAGTCAGAT-3′, negative control (shNC): 5′-TTCTCCGAACGTGTCACGTTT-3′.

### Immunohistochemistry and immunofluorescence

For immunohistochemistry (IHC) as previously stated, 4 µm paraffin sections were deparaffinized and rehydrated17. Antigen retrieval involved heating in a citrate buffer (pH 6.0) using a microwave for 20 min. Endogenous peroxidase activity was inhibited by treating with 3% hydrogen peroxide at room temperature for 10 min after cooling. Sections were blocked with 5% BSA in PBS for 1 h to reduce nonspecific binding. Primary antibodies were incubated overnight at 4 °C. The following day, HRP-conjugated secondary antibodies (diluted 1:500) were applied and incubated at room temperature for 1 h, then developed with DAB. Sections were stained with hematoxylin–eosin and mounted with coverslips. Cells were placed on poly-lysine-coated slides for immunofluorescence (IF). Cells at 70% confluence were fixed with 4% paraformaldehyde for 20 min, washed with PBS, permeabilized using 0.1% Triton X-100 for 10 min, and blocked with 1% BSA/PBS for 30 min. Primary antibodies were incubated overnight, followed by staining with fluorescently labeled secondary antibodies the next day. Nuclei were DAPI-stained. (USP14, 14,517-1-AP, Proteintech, diluted 1:100-1:500; BAG4, 13,913-1-AP, Proteintech, diluted 1:500-1:1000; PARKIN, 14,060-1-AP, Proteintech, diluted 1:200-1:800).

### CCK8, EDU and colony formation assay

In the CCK-8 assay, 2000 cells were plated per well in a 96-well plate and cultured for 24–72 h. Subsequently, 10 µL of CCK-8 reagent was added to each well and incubated for 1 h at 37 °C in 5% CO_2_. Absorbance was measured at 450 nm using a microplate reader, and a proliferation curve was plotted based on the absorbance values (Gaudio et al. 2022). For the EDU assay, EdU working solution (10 μM) was added to the culture medium and incubated for 2 h. Cells were fixed with 4% paraformaldehyde for 15–20 min, permeabilized using 0.5% Triton X-100 for 10 min, and then subjected to a 30-min Click reaction with a fluorescent probe. Nuclei were stained with DAPI, and fluorescence was detected using a microscope or flow cytometer (Jing et al. 2023). In the colony formation assay, 1000 cells were seeded per well in a 6-well plate and cultured for 1–2 weeks, with regular medium changes. After colony formation, cells were fixed with 4% paraformaldehyde for 15 min, stained with 0.1% crystal violet for 30 min, and washed with water. Colonies were then counted, and the colony formation rate was calculated (Zhang et al. 2022b).

### Co-IP and western blot

When the cells reached 80% confluence, they were lysed for 30 min with lysis buffer containing 1% NP-40, 150 mM NaCl, 50 mM Tris–HCl pH 7.4, 1 mM EDTA, and a protease inhibitor cocktail. Insoluble material was removed by centrifugation (14,000 g, 10 min, 4 °C). For immunoprecipitation, 1 mg of protein lysate was taken and 2 µg of specific antibody was added, followed by gentle shaking overnight at 4 °C. The next day, 30 µL of pre-washed Protein A/G beads (MCE) were added to the sample and gently shaken for 4 h to facilitate binding of the antibody-antigen complex to the beads. Beads were collected by low-speed centrifugation (1000 g, 3 min, 4 °C) and washed three times with PBS. The protein was transferred from the gel to a PVDF membrane (using wet transfer, 100 V, 1 h). The membrane was blocked with 5% non-fat milk, then incubated overnight at 4 °C with a specific primary antibody. Anti-USP14 antibody (14,517-1-AP, Proteintech), anti-TOM20 antibody (11,802-1-AP, Proteintech), anti-TOM22 antibody (11,278-1-AP, Proteintech), anti-LC3B antibody (14,600-1-AP, Proteintech), anti-BAG4 antibody (13,913-1-AP, Proteintech), anti-ubiquitin antibody (10,201-2-AP, Proteintech), anti-PARP1 antibody (13,371-1-AP, Proteintech), anti-BCL2 antibody (12,789-1-AP, Proteintech), anti-BAX antibody (50,599-2-Ig, Proteintech), anti-Parkin antibody (14,060-1-AP, Proteintech), anti-β-actin antibody (20,536-1-AP, Proteintech), anti-Tubulin antibody (11,224-1-AP, Proteintech), anti-COXIV antibody (11,242-1-AP, Proteintech), anti-flag antibody (20,543-1-AP, Proteintech), anti-myc tag antibody (16,286-1-AP, Proteintech), anti-HA tag antibody (51,064-2-AP, Proteintech). The next day, it was incubated with an HRP-conjugated secondary antibody (diluted 1:5000) for 1 h and the signal was developed using the ECL detection system.

### RNA extraction and quantitative PCR (qPCR)

Total RNA was extracted using TRIzol reagent (Invitrogen) following the manufacturer’s instructions. Briefly, 1 mL of TRIzol was added to each dish to lyse the cells, then 0.2 mL of chloroform was added for phase separation. Following centrifugation, the aqueous phase was collected, and RNA was precipitated with an equal volume of isopropanol. The RNA pellet was washed with 75% ethanol, centrifuged, dried, and dissolved in RNase-free water. RNA concentration and purity were measured using a NanoDrop spectrophotometer, ensuring an A260/A280 ratio between 1.8 and 2.0. cDNA was synthesized from 1 µg of RNA using a reverse transcription kit (Qiagen, Germany). qPCR was performed with SYBR Green PCR Master Mix (Applied Biosystems). Each 20 µL reaction contained 10 µL of SYBR Green mix, 1 µL of forward and reverse primers (200 nM), 2 µL of cDNA, and water. The cycling conditions were: 95 °C for 2 min for pre-denaturation, followed by 40 cycles of 95 °C for 15 s and 60 °C for 1 min. All reactions were run in triplicate. Relative gene expression was calculated using the ΔΔCt method, with β-actin as the internal control. Human BAG4 primer: 5′-CTCTTCGCCCTGAACCTC-3′ (forward), 5′-GGACCATACGCTCCATTT-3′ (reverse); Human β-actin primer: 5′-ATTGCCGACAGGATGCAGAA-3′ (forward), 5′-GCTGATCCACATCTGCTGGAA-3′ (reverse). Data analysis was done using GraphPad Prism.

### Mitophagy assay using AAV-COX8-EGFP-mCherry reporter

To monitor mitophagy in live cells, we utilized an AAV-mediated COX8-EGFP-mCherry fluorescence reporter system, which enables visualization of mitochondrial degradation in lysosomes. The COX8 domain ensures mitochondrial targeting, while the dual fluorophores: EGFP and mCherry, allow differential detection of mitochondrial integrity and mitophagy progression based on their pH stability. Cells were infected with AAV-COX8-EGFP-mCherry (HANBIO, China) at a multiplicity of infection (MOI) of 5 and incubated for 48 h to allow stable expression of the reporter protein. Following infection, cells were treated under appropriate mitophagy-inducing conditions. Live-cell imaging was performed using a Zeiss LSM 780 confocal microscope equipped with oil immersion objectives. Excitation/emission wavelengths were set to 488 nm (EGFP) and 561 nm (mCherry). The system functions as follows: EGFP is pH-sensitive and quenched upon mitophagy when mitochondria are degraded in lysosomes. mCherry is pH-stable, retaining red fluorescence even in acidic lysosomal environments. In non-mitophagic conditions, mitochondria exhibit a yellow (merged EGFP/mCherry) signal. Upon mitophagy induction, EGFP fluorescence is lost due to lysosomal acidification, leaving red-only puncta, indicative of mitochondrial degradation.

### Mitochondrial protein extraction

Cells were collected after washing and treatment with 0.25% trypsin in PBS, followed by mitochondrial isolation using Mitochondria Isolation Kit (Beyotime Biotechnology). Cells were resuspended in a lysis buffer (250 mM sucrose, 1 mM EGTA, pH 7.4-adjusted mannitol) and homogenized 25 times with a Dounce homogenizer. The homogenate was centrifuged at 700 g for 10 min to remove unbroken cells and debris. The supernatant was then centrifuged at 10,000 g for 25 min to isolate the mitochondria. The mitochondrial pellet was washed and resuspended in lysis buffer containing 1% Triton X-100, 50 mM Tris–HCl (pH 7.4), 150 mM NaCl, 1 mM EDTA, and protease inhibitors. The mixture was gently shaken for 1 h to ensure complete lysis, then centrifuged at 15,000 g for 15 min to collect the protein-containing supernatant.

### Flow cytometric apoptosis analysis

Annexin V and PI double staining method, collecting cells from the culture dish, including both suspended and adherent cells. Collect cells by centrifugation and discard the supernatant. Resuspend the cells in buffer and centrifuge to remove any residuals from the culture medium. Repeat the washing step to ensure cells are clean. Resuspend the cells in 100 μL of Annexin V binding buffer. Add 5 μL of Annexin V-FITC and 5 μL of PI. After gently mixing, incubate in the dark at room temperature for 15 min. After staining, add 400 μL of buffer to dilute the cell suspension. Analyze using a flow cytometer, setting up channels to detect the FITC and PI signals separately. In data analysis, live cells that have not undergone apoptosis are generally negative for both Annexin V and PI. Early apoptotic cells are characterized by being Annexin V positive and PI negative. Cells in late apoptosis or necrosis exhibit positivity for both Annexin V and PI.

### BALB/c nude mice tumor xenograft model

Six BALB/c nude mice for each group were used for xenograft model. Culture LoVo cells at 37 °C, 5% CO₂. After collecting the cells, wash them with PBS and count them, adjusting the cell concentration to 1 × 10^7 cells/mL (Tang et al. 2020). Disinfect the right flank of the mouse with an alcohol swab. After anesthetizing the mouse, use a 1 mL syringe to subcutaneously inject 100 μL of the cell suspension (approximately 1 × 10^6 cells). Monitor and record tumor growth: Measure the three-dimensional size of the tumor every 4 days with calipers. Calculate the tumor volume: Volume = (length × width2)/2 (Li et al. 2014). All animal experiments were conducted in accordance with the guidelines approved by the Institutional Animal Care and Use Committee (IACUC) and complied with the ethical principles for animal research. Efforts were made to minimize animal suffering and reduce the number of animals used.

### Statistical analysis

Statistical analyses were conducted using GraphPad Prism 8.3.0. Continuous data were presented as mean ± standard deviation and analyzed using Student’s *t*-test or ANOVA. Kaplan–Meier curves were used for survival analysis, with significance assessed via the log-rank test. A significance level of *p* < 0.05 was established.

## Results

### DUBs library screen reveals USP14 as a mitophagy regulator in CRC

**Fig. 1:**
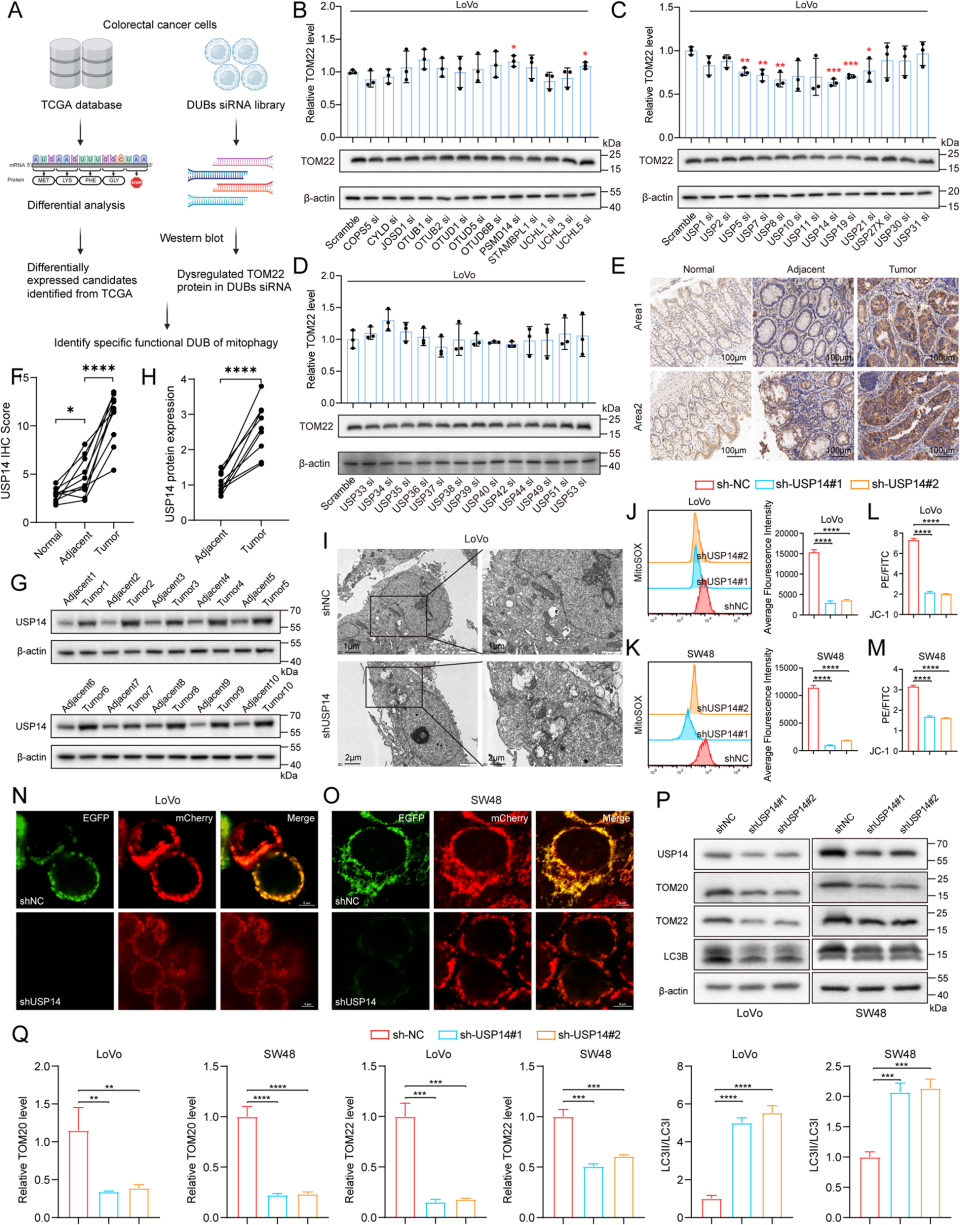
USP14 is a potential mitophagy regulator in CRC (MSI-H). Scheme displaying the procedure used for identifying mitophagy regulator **a**. Protein expression levels of TOM22 in LoVo cells with 41 kinds of deubiquitinases knocked down, respectively **b**–**d**. Immunohistochemical (IHC) staining of USP14 in 10 paired of tumor and adjacent non-tumor tissues, along with statistical quantification results **e** and **f**. Western Blot of USP14 protein expression in tumor and adjacent non-tumor tissues, along with statistical quantification results **g** and **h**. Representative images from transmission electron microscopy showing autophagosomes in LoVo cells after knocking down USP14 **i**. Flow cytometry analysis of MitoSOX-Red and average fluorescence intensity **j** and **k**. Quantification of JC-1 staining **l** and **m**. Mitophagy was quantified using the Cox8-EGFP-mCherry marker **n** and **o**. Mitophagy related markers were detected by Western Blot **p** and **q**. *P < 0.05, **P < 0.01, ***P < 0.001, ****P < 0.0001. Data are presented as the mean ± SD of three separate experiments

Mitophagy is critically implicated in tumor progression (Liu et al. 2024). Emerging evidence indicates that mitophagy exerts divergent roles across different tumor types (Wang et al. 2014). In CRC, mitophagy has been shown to suppress tumor proliferation, yet the precise molecular mechanisms governing its regulation remain poorly understood. Ubiquitination, a crucial post-translational modification involving ubiquitin, regulates of protein stability, localization, and function, with deubiquitinating enzymes (DUBs) being key to these processes. Based on our findings, we hypothesize that a specific DUB may play a crucial role in regulating mitophagy in CRC. To explore this hypothesis, we analyzed the expressions of 73 DUBs in publicly available datasets and identified 41 DUBs that exhibit differential expression between CRC tissues and adjacent normal tissues (Fig. S1). To further investigate the role of these DUBs, we employed an siRNA library screen targeting these candidates and assessed the impact on the mitophagy marker TOM22 (Fig. 1a). Our results demonstrated that knockdown of USP5, USP7, USP8, USP14, USP19, USP21 led to a significant reduction in TOM22 expression in the LoVo CRC cell line, while knockdown of PSMD14 and UCHL5 led to a significant increase (Fig. 1b–d). siPSMD14 and siUCHL5 lead to increase in TOM22, which means suppression of mitophagy and pro-tumor effect, but they are highly expressed in tumor. siUSP7, siUSP8, siUSP14 and siUSP19 lead to the largest reduction in TOM22, which means induction of mitophagy and anti-tumor effect, but USP8 and USP19 are low expressed in tumor. The above results suggest a reliable potential regulatory role of USP7 and USP14 in mitophagy. Since mitophagy occurs in the cytoplasm while USP7 is localized in the nucleus, we chose to further investigate USP14, which is localized in the cytoplasm. To corroborate these findings, we conducted immunohistochemical and western blot analysis on tumor samples. The analysis demonstrated a significant disparity in USP14 expression between tumor and adjacent non-tumor tissues, underscoring USP14’s differential expression and warranting its selection for further investigation (Fig. 1e–h). Transmission electron microscopy revealed an increased abundance of autophagosomes in cells with USP14 knockdown, further suggesting an enhancement in mitophagy (Fig. 1i). Silencing USP14 significantly reduced mitochondrial reactive oxygen species (ROS) levels and mitochondrial membrane potential, highlighting disruptions in mitochondrial function (Fig. 1j–m). To provide more direct evidence supporting the role of USP14 in mitophagy, we injected adenovirus that expresses the mitophagy biosensor EGFP-mCherry-COX8 into the CRC cells. While USP14 knockdown induced a severe mitophagy, mitochondria represented by the mCherry signal were markedly lower while fluorescence from EGFP is quenched (Fig. 1n, o). Western blot analysis revealed that the knockdown of USP14 resulted in a significant reduction in the levels of mitochondrial markers TOM20 and TOM22, while LC3B-II/LC3B-I levels were increased (Fig. 1p, q). Hence, we identify USP14 as a pivotal regulator of mitophagy in CRC. These findings not only elucidate the role of USP14 in mitophagy but also suggest its potential as a novel therapeutic target for CRC treatment.

### USP14 promotes CRC (MSI-H) tumorigenesis and oxaliplatin resistance in vitro and vivo

**Fig. 2:**
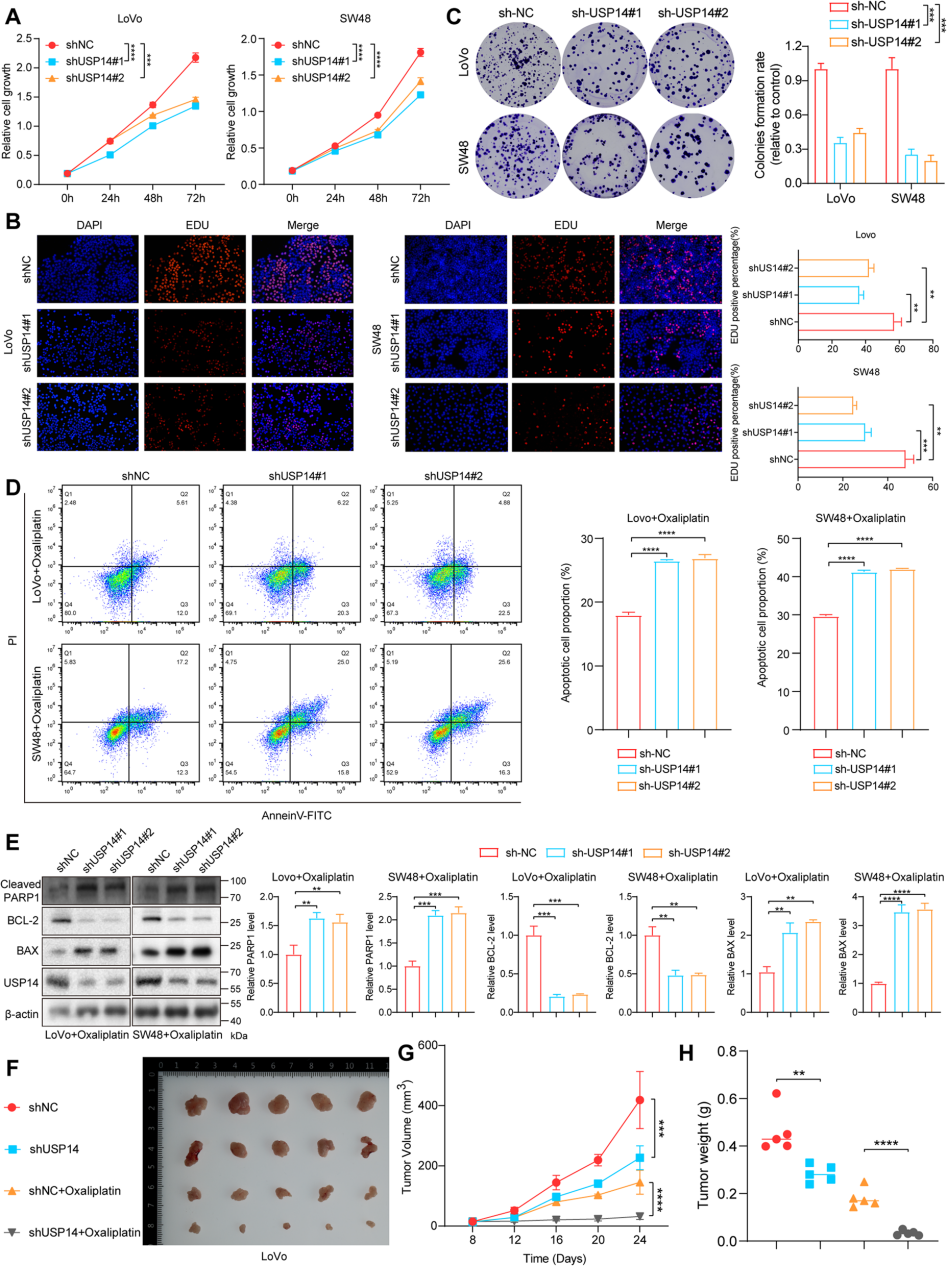
USP14 promotes CRC (MSI-H) tumorigenesis and oxaliplatin resistance. CCK8 detects cell proliferation activity **a**. Image and quantitative analysis of cell cloning and formation **b**. The EdU staining results and quantification of different groups **c**. The apoptotic cells were measured by flow cytometry and analyzed by flow Jo **d**. The apoptosis-associated markers were evaluated by Western blot **e**. Measurement of the role of USP14 knockdown on the subcutaneous xenograft tumors from LoVo cells **f**, tumor volume **g** and tumor weight **h** were shown. *P < 0.05, **P < 0.01, ***P < 0.001, ****P < 0.0001. Data are presented as the mean ± SD of three separate experiments

Given the aberrant expression of USP14 in CRC (MSI-H), we conducted a comprehensive study to examine its role in tumor progression and sensitivity to chemotherapy. CCK8 assays demonstrated that knockdown of USP14 led to a significant reduction in cell viability by 37% (LoVo) and 32% (SW48) at 72h, suggesting its critical role in supporting cancer cell proliferation (Fig. 2a). USP14 knockdown significantly reduced cancer cell proliferation by 20% (LoVo) and 25% (SW48) and clonogenic potential by 60% (LoVo) and 69% (SW48), as confirmed by colony formation and EDU assays (Fig. 2b, c). These results collectively highlight the essential role of USP14 in maintaining cancer cell growth. We investigated USP14’s impact on cancer cell sensitivity to the chemotherapeutic agent oxaliplatin. Upon USP14 knockdown, cancer cells exhibited a significantly enhanced response to oxaliplatin treatment, as evidenced by a marked increase in apoptosis by 37% (LoVo) and 25% (SW48) (Figs. 2d and S2a, b). Flow cytometry apoptosis assays and Western blot analysis of apoptotic markers demonstrated increased apoptosis in USP14-depleted cells exposed to oxaliplatin (Li et al. 2014). The knockdown of USP14 induced an upregulation of PARP1 and BAX, proteins associated with apoptotic cell death, and a concomitant downregulation of the anti-apoptotic protein Bcl2 (Fig. 2e). This indicates that the enhanced sensitivity to oxaliplatin observed in USP14-deficient cells is mediated, at least in part, through the modulation of apoptosis pathways. To extend these findings to an in vivo context, we employed xenograft models in which cancer cells with USP14 knockdown were subcutaneously injected into nude mice. The results mirrored our in vitro findings, with the USP14-depleted tumors exhibiting significantly slower growth rates compared to the control group through Ki67 IHC staining (Fig. S2c, d). More importantly, these USP14-knockdown tumors showed a pronounced increase in sensitivity to oxaliplatin treatment by TUNNEL staining (Fig. S2e). Mice bearing USP14-deficient tumors responded more robustly to oxaliplatin, with a substantial reduction in both tumor size and weight by approximately 32% and 67% (oxaliplatin treatment), as compared to control tumors (Figs. 2f–h and S2d). These results suggest that USP14 not only drives tumor progression but also plays a key role in modulating chemosensitivity in cancer.

### USP14 interacts with BAG4

**Fig. 3:**
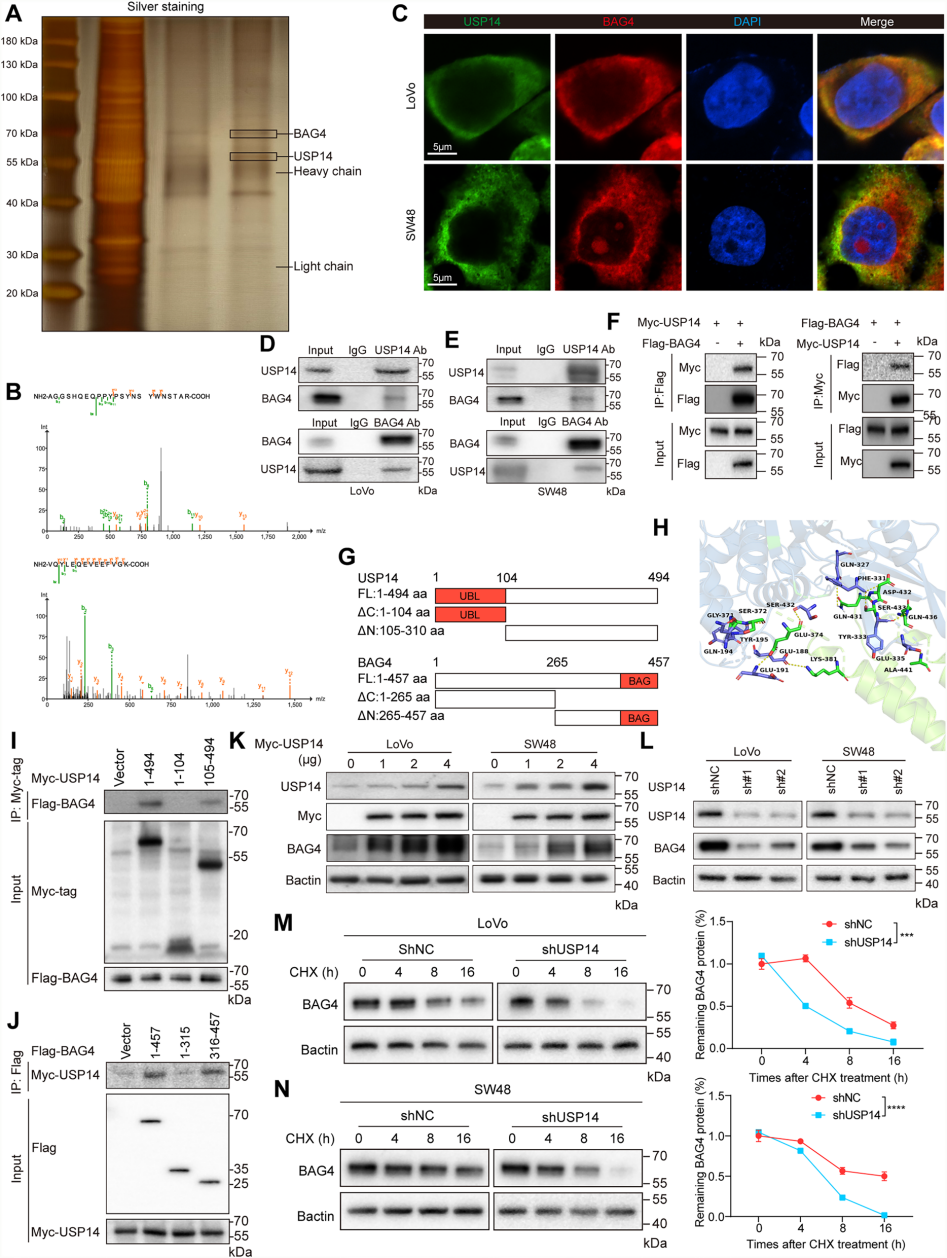
USP14 interacts with BAG4. Silver-stained SDS-PAGE gel from IgG and USP14 purifications **a**. Secondary mass spectrum of USP14 and BAG4 **b**. Immunofluorescence staining presented the co-localization of USP14 and BAG4 in CRC (MSI-H) **c**. Co-IP demonstrates the endogenous interaction between USP14 and BAG4 in LoVo and SW48 cells **d** and **e**. Co-IP demonstrates the exogenous interaction between USP14 and BAG4 in 293 T cells **f**. Design of USP14 and BAG4 truncations **g**. 2D diagram of molecular docking **h**. Immunoprecipitation and WB analyses showing the interactions between FLAG-tagged truncated BAG4 and Myc-tagged USP14 proteins in HEK293T cells **i**. Immunoprecipitation and WB analyses showing the interactions between FLAG-tagged BAG4 proteins and Myc-tagged truncated USP14 in HEK293T cells **j**. Western blotting analyses and quantification of the indicated cells transfected with USP14 plasmids and USP14 shRNA **k** and **l**. CHX chase analysis of BAG4 protein half-life in USP14-kncokdown and control group in CRC (MSI-H) (**m** and ** n**). *P < 0.05, **P < 0.01, ***P < 0.001, ****P < 0.0001. Data are presented as the mean ± SD of three separate experiments

To further elucidate the molecular mechanisms by which USP14 regulates mitophagy, we initially performed immunoprecipitation (IP) assays using USP14-specific antibodies, followed by silver staining and mass spectrometry. This analysis identified several USP14-interacting proteins (Fig. 3a, b), including BAG4, a protein involved in mitophagy, which we chose for detailed investigation. Using immunofluorescence in LoVo and SW48 cells, we observed significant cytoplasmic colocalization of USP14 and BAG4 (Fig. 3c), suggesting a functional relationship. To confirm the physical interaction, co-immunoprecipitation (Co-IP) assays were performed, and the results confirmed the direct binding between USP14 and BAG4 in both cancer cell lines (Fig. 3d, e). Further validation of the USP14-BAG4 interaction was obtained by exogenous Co-IP assays in HEK293T cells, where Myc-tagged USP14 and Flag-tagged BAG4 plasmids were co-transfected. The Co-IP results showed that USP14 effectively bound BAG4 in this system (Fig. 3f). To map the specific domains responsible for their interaction, we constructed truncated mutants of USP14 and BAG4 (Fig. 3g). Molecular docking analysis further identified several potential binding interfaces between USP14 and BAG4 at the amino acid level (Fig. 3h). The results identified a specific domain within USP14 that is critical for binding BAG4 (Fig. 3i), while a specific region of BAG4 was also pinpointed as essential for the interaction (Fig. 3j). These findings highlight the structural specificity of the USP14-BAG4 interaction and suggest a mechanism by which USP14 modulates BAG4 activity in cells. We then explored the functional consequences of this interaction by examining BAG4 stability. Overexpression of USP14 caused a dose-dependent increase in BAG4 protein (Fig. 3k), while knockdown of USP14 in cancer cells resulted in a marked reduction in BAG4 protein levels (Fig. 3l). Interestingly, despite significant changes in protein levels, no alterations were observed at the mRNA level, suggesting that USP14 regulates BAG4 primarily at the post-transcriptional level. Further confirmation of USP14’s role in stabilizing BAG4 came from cycloheximide (CHX) chase assays. These experiments revealed that BAG4 protein exhibited a significantly shortened half-life in USP14-knockdown cells, further implicating USP14 in the post-translational stabilization of BAG4 (Fig. 3m, n). Together, these results strongly indicate that USP14 is essential for maintaining BAG4 protein stability through its deubiquitinase activity. In summary, this study uncovers a novel regulatory axis involving USP14 and BAG4 in cancer cells. USP14 directly interacts with BAG4 and modulates its stability, thereby contributing to the regulation of mitophagy.

### USP14 mediates K48-deubiquitination of BAG4 at K403

**Fig. 4:**
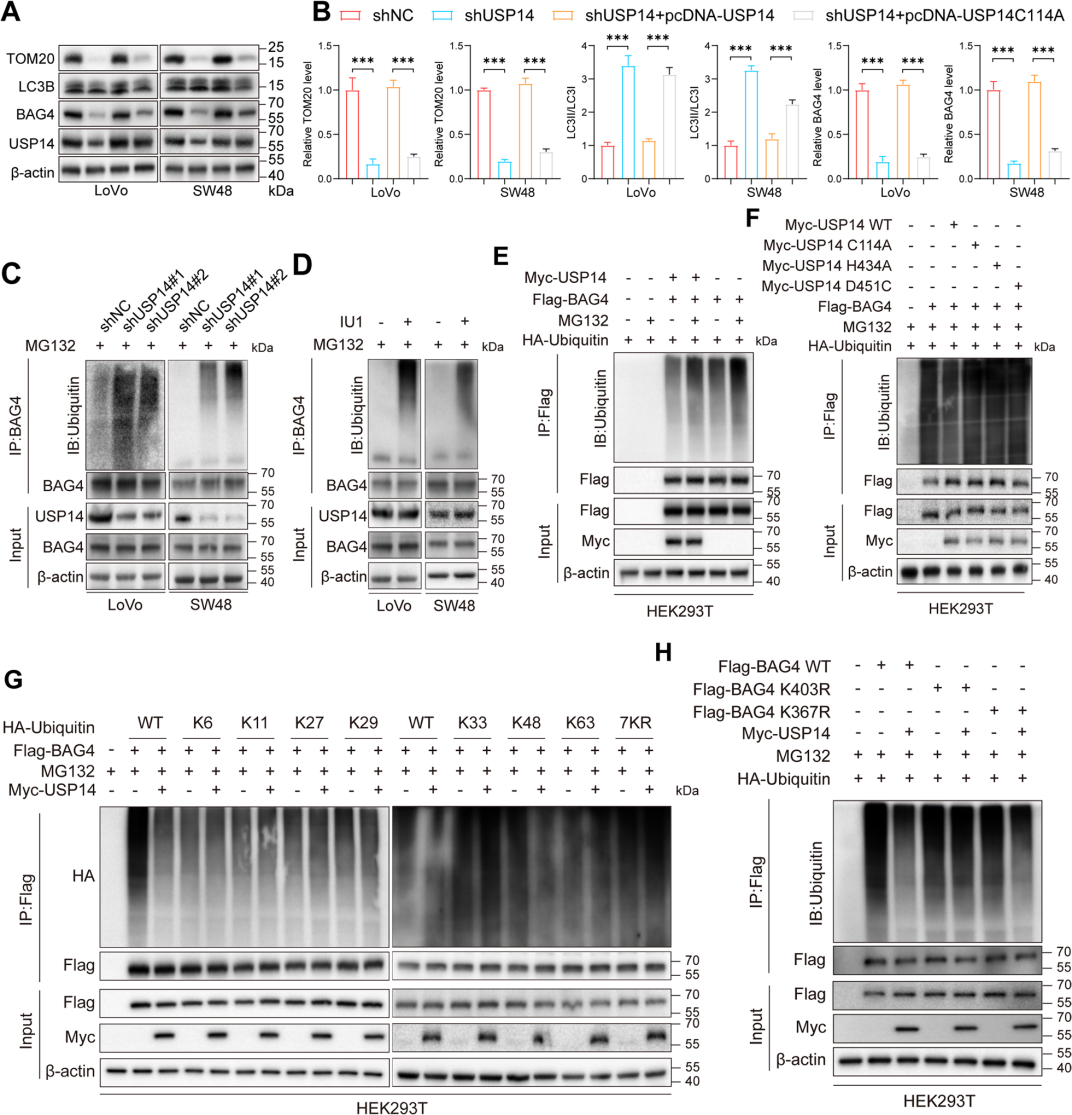
USP14 mediates K48-deubiquitination of BAG4 at K403. WB analysis showed the BAG4 expression and mitophagy markers after overexpressing USP14 WT and USP14 C114A in USP14-knockdown cells **a** and **b**. Analysis of BAG4 ubiquitination by denatured-IP in USP14-knockdown cells **c** and IU1-treated cells **d**. HEK293T cells were transfected with Flag-BAG4, HA-ubiquitin, and Myc-USP14 (wild type and deubiquitinase-deficient mutants) for 2 days. BAG4 was immunoprecipitated and probed for ubiquitin **e** and **f**. The levels of different K-linked BAG4 ubiquitination upon Flag-USP14 transfection were assessed by Western blotting using an anti-HA antibody **g**. HEK293T cells were transfected with Flag-BAG4 (WT), Flag-BAG4 (K367R), and Flag-BAG4 (K403R) with or without Myc-USP14, to examine changes in ubiquitination **h**. *P < 0.05, **P < 0.01, ***P < 0.001, ****P < 0.0001. Data are presented as the mean ± SD of three separate experiments

The findings indicate that USP14 is essential for BAG4 stabilization via its deubiquitination function. To verify whether USP14 deubiquitination activity is crucial for the changes in BAG4 expression and mitophagy, we conducted experiments using the catalytic-inactive form of USP14 overexpression (USP14 C114A). The results showed USP14 WT overexpression, but not USP14 C114A, could rescue the changes in BAG4 expression and mitophagy after knocking down USP14 (Fig. 4a, b). Knockdown of USP14 or treatment with a USP14-specific inhibitor (IU1) (Fig. S3a) led to significantly increased BAG4 ubiquitination levels in cancer cells (Fig. 4c, d), implying that USP14 normally functions to deubiquitinate BAG4 and prevent its degradation. To further confirm this hypothesis, we co-transfected HEK293T cells with constructs encoding BAG4-3 × Flag, HA-ubiquitin, and Myc-USP14, while using MG132 to inhibit proteasomal degradation. Immunoprecipitation and Western blot analysis with an anti-HA antibody revealed that USP14 markedly decreased BAG4 polyubiquitination (Fig. 4e), further supporting the role of USP14 in deubiquitinating and stabilizing BAG4. This finding was reinforced by the use of deubiquitinase-deficient USP14 mutants (C114A, H434A, and D451C), all of which failed to rescue BAG4 from ubiquitination, suggesting that USP14’s enzymatic activity is essential for this process (Fig. 4f). We employed HA-tagged ubiquitin mutants targeting specific linkage types (K6, K11, K27, K29, K33, K48, K63, and 7KR) in ubiquitination assays to determine the ubiquitin chain types attached to BAG4. The results revealed that BAG4 is primarily modified by K48-linked ubiquitin chains (Fig. 4g), which are typically associated with proteasomal degradation, underscoring the protective role of USP14 against BAG4 degradation. Further, to determine the precise deubiquitination sites on BAG4, we identified two potential ubiquitination sites (K367 and K403) and generated lysine-to-arginine (KR) mutant plasmids. Ubiquitination assays showed that mutating the K403 site (K403R) significantly reduced BAG4 ubiquitination in the presence of USP14, whereas mutations at other lysine sites, such as K367, had minimal impact (Fig. 4h). These results indicate that USP14 stabilizes BAG4 by specifically deubiquitinating K48-linked ubiquitin chains at the K403 residue. In summary, this study establishes that USP14 promotes the stability of BAG4 by directly removing K48-linked ubiquitin chains at the K403 site, thereby protecting BAG4 from proteasomal degradation. Lastly, a protein–protein interaction assay comparing USP14 WT, USP14 inactive mutant (C114A) and BAG4 were performed. Our results demonstrate that the interaction between USP14 and BAG4 is significantly reduced in the C114A mutant, indicating that USP14 deubiquitinating activity is necessary for stabilizing BAG4 (Fig. S3b). This process highlights a key mechanism through which USP14 controls BAG4 protein stability in CRC (MSI-H), offering insights into the USP14-BAG4 axis’s role in tumor biology and suggesting a potential therapeutic target for disrupting BAG4.

### USP14 restrains parkin recruitment into mitochondria through BAG4

**Fig. 5:**
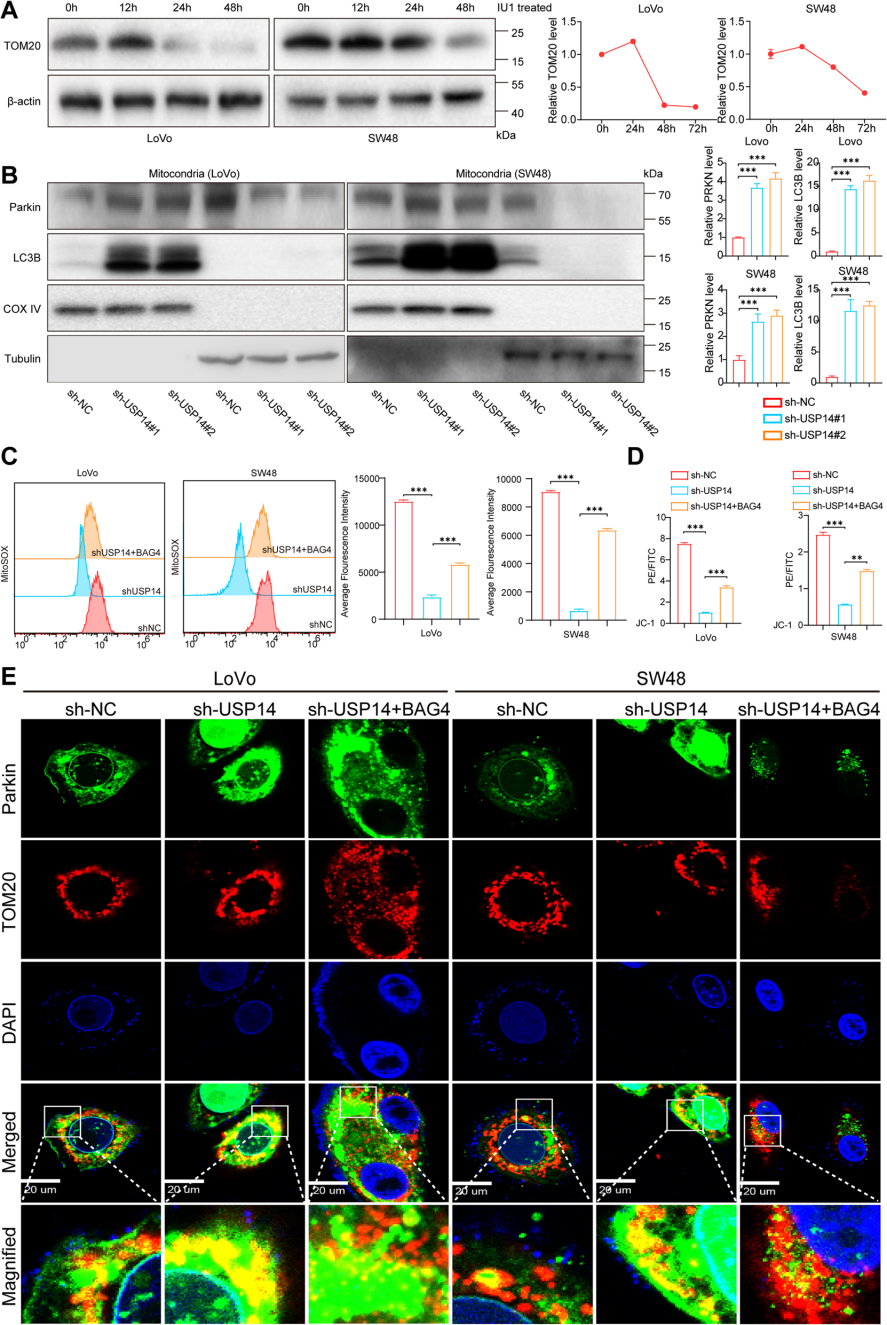
USP14 restrains Parkin recruitment into mitochondria through BAG4. Western blotting analysis of TOM20 after treating cells for 0, 12, 24, and 48 h. With USP14 inhibitor **a**. Western blotting analysis of Parkin and LC3 expression in mitochondria isolated from indicated cells **b**. Flowcytometry analysis of MitoSOX-Red and average fluorescence intensity after rescuing BAG4 expression in USP14-knockdown cells **c**. Quantification of JC-1 staining after rescuing BAG4 expression in USP14-knockdown cells **d**. Representative localization of PRKN (green) relative to TOM20-positive (red) mitochondria **e**. *P < 0.05, **P < 0.01, ***P < 0.001, ****P < 0.0001. Data are presented as the mean ± SD of three separate experiments

Firstly, we conducted a time-course experiment using a USP14 inhibitor, treating cells for 0, 12, 24, and 48 h. We then assessed mitophagy by detecting TOM20 at these time points. Our results further confirm that USP14 inhibition induces mitophagy in a time-dependent manner (Fig. 5a). BAG4 is recognized as a key negative regulator of Parkin (PRKN), primarily by directly interacting with and preventing its recruitment to depolarized mitochondria. Based on this relationship, we propose that USP14 inhibits mitophagy in CRC (MSI-H) via the BAG4/PRKN axis. To evaluate the hypothesis, mitochondrial proteins were extracted and analyzed from isolated mitochondria of USP14-knockdown and control cancer cell lines. Western blot results indicated a significant increase in mitochondrial PRKN levels upon USP14 knockdown, accompanied by elevated LC3B protein levels in both LoVo and SW48 cells (Fig. 5b), suggesting enhanced mitophagy. To further investigate the involvement of BAG4 in USP14-mediated inhibition of mitophagy, we performed rescue experiments by restoring BAG4 expression in USP14-knockdown cells. Notably, both mitochondrial ROS levels and mitochondrial membrane potential (MMP), which were disrupted in USP14-deficient cells, were restored upon BAG4 reintroduction (Fig. 5c, d). The findings indicate that BAG4 is essential for mitochondrial homeostasis through its regulation of ROS production and MMP. In addition, we used immunofluorescence staining to label mitochondria with TOM20 and Parkin to assess their colocalization, a hallmark of mitophagy activation (Fig. 5e). USP14 depletion significantly increased the colocalization of Parkin with mitochondria, indicating enhanced mitophagy, while BAG4 restoration partially reversed this effect, reducing Parkin’s mitochondrial recruitment. The findings indicate that USP14 stabilizes BAG4, which restricts Parkin recruitment to mitochondria and consequently suppresses mitophagy in cancer cells. This mechanism provides valuable insight into how USP14 may regulate mitochondrial dynamics and autophagy, potentially contributing to the survival and progression of cancer cells. USP14 may hinder mitophagy, resulting in the accumulation of damaged mitochondria, which can drive metabolic reprogramming and increase chemosensitivity, key factors in cancer progression and therapy resistance. Targeting the USP14/BAG4/PRKN axis to enhance mitophagy shows promise for improving cancer treatment efficacy.

### USP14-triggered tumorigenesis and oxaliplatin resistance are mediated by BAG4

**Fig. 6:**
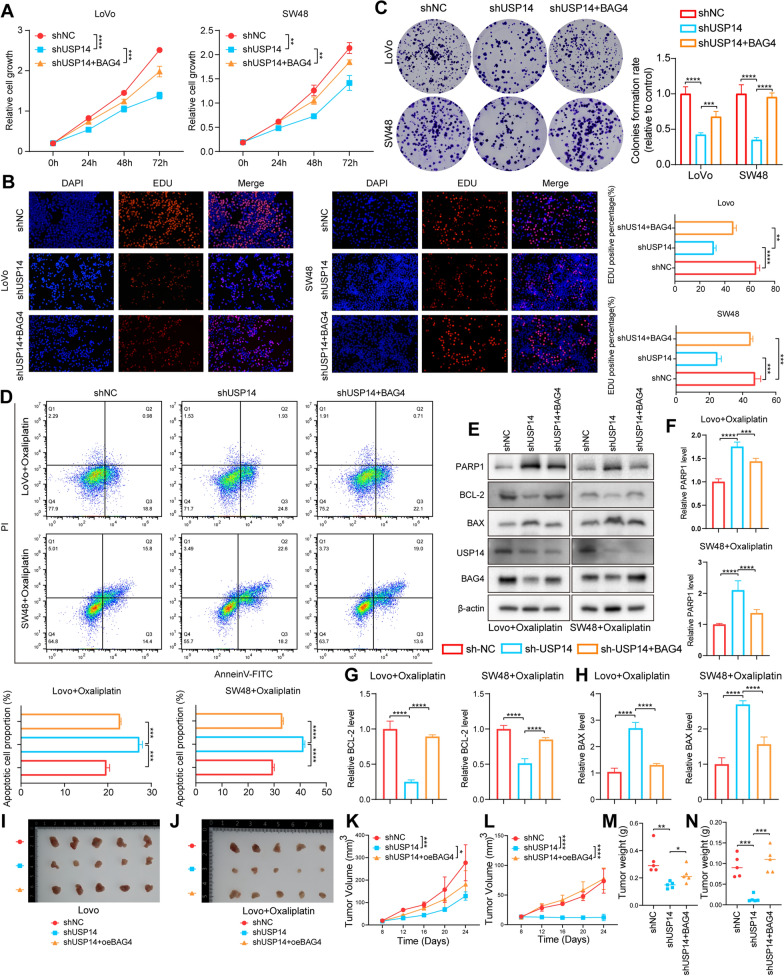
USP14-triggered tumorigenesis and oxaliplatin resistance are mediated by BAG4. CCK8 detects cell proliferation activity **a**. The EdU staining results and quantification of different groups **b**. Image and quantitative analysis of cell cloning and formation **c**. The apoptotic cells were measured by flow cytometry and analyzed by flow Jo **d**. The apoptosis-associated markers were evaluated by Western blot **e**. Quantification of apoptosis-associated markers **f**–**h**. Measurement of the role of USP14 knockdown on the subcutaneous xenograft tumors from LoVo cells **i** and **j**, tumor volume **k** and **l** and tumor weight **m** and **n** were shown. *P < 0.05, **P < 0.01, ***P < 0.001, ****P < 0.0001. Data are presented as the mean ± SD of three separate experiments

To further investigate the dependency of CRC (MSI-H) progression and chemosensitivity on BAG4 mediated by USP14, we simultaneously upregulated BAG4 in USP14-knockdown SW48 and LoVo cell lines. Results from CCK8 (Fig. 6a) and EDU (Fig. 6b) assays revealed that silencing USP14 significantly suppressed cancer cell proliferation, while BAG4 overexpression rescued this effect. Similarly, colony formation assays indicated that USP14 knockdown impaired the clonogenic ability of cancer cells, which was restored by BAG4 overexpression (Fig. 6c). To explore BAG4’s impact on USP14-mediated sensitivity to oxaliplatin, we conducted flow cytometry to assess apoptosis and performed Western blot (WB) analysis of apoptosis-related proteins. Flow cytometry showed that overexpression of BAG4 almost completely reversed the increased oxaliplatin sensitivity induced by USP14 knockdown (Fig. 6d). Consistent with this observation, WB analysis demonstrated that BAG4 overexpression attenuated USP14 knockdown-induced changes in apoptosis markers, specifically the upregulation of PARP1 and BAX, and the downregulation of Bcl2 (Fig. 6e–h). Supported by these in vitro findings, we extended our investigation to an in vivo mouse xenograft model, where cancer cells were subcutaneously implanted into nude mice. Consistent with our in vitro data, knockdown of USP14 significantly suppressed tumor growth, while BAG4 overexpression abrogated this inhibitory effect (Figs. 6i–m and S2d). Moreover, in the context of oxaliplatin treatment, USP14 knockdown markedly enhanced drug sensitivity, an effect that was also reversed by BAG4 overexpression (Figs. 6j–n and S2f–h). In conclusion, the data indicate that BAG4 is crucial in USP14-driven tumorigenesis and chemosensitivity in CRC, significantly influencing cancer progression and oxaliplatin treatment response.

### The clinical relevance of USP14, BAG4, and PRKN in CRC (MSI-H)

**Fig. 7:**
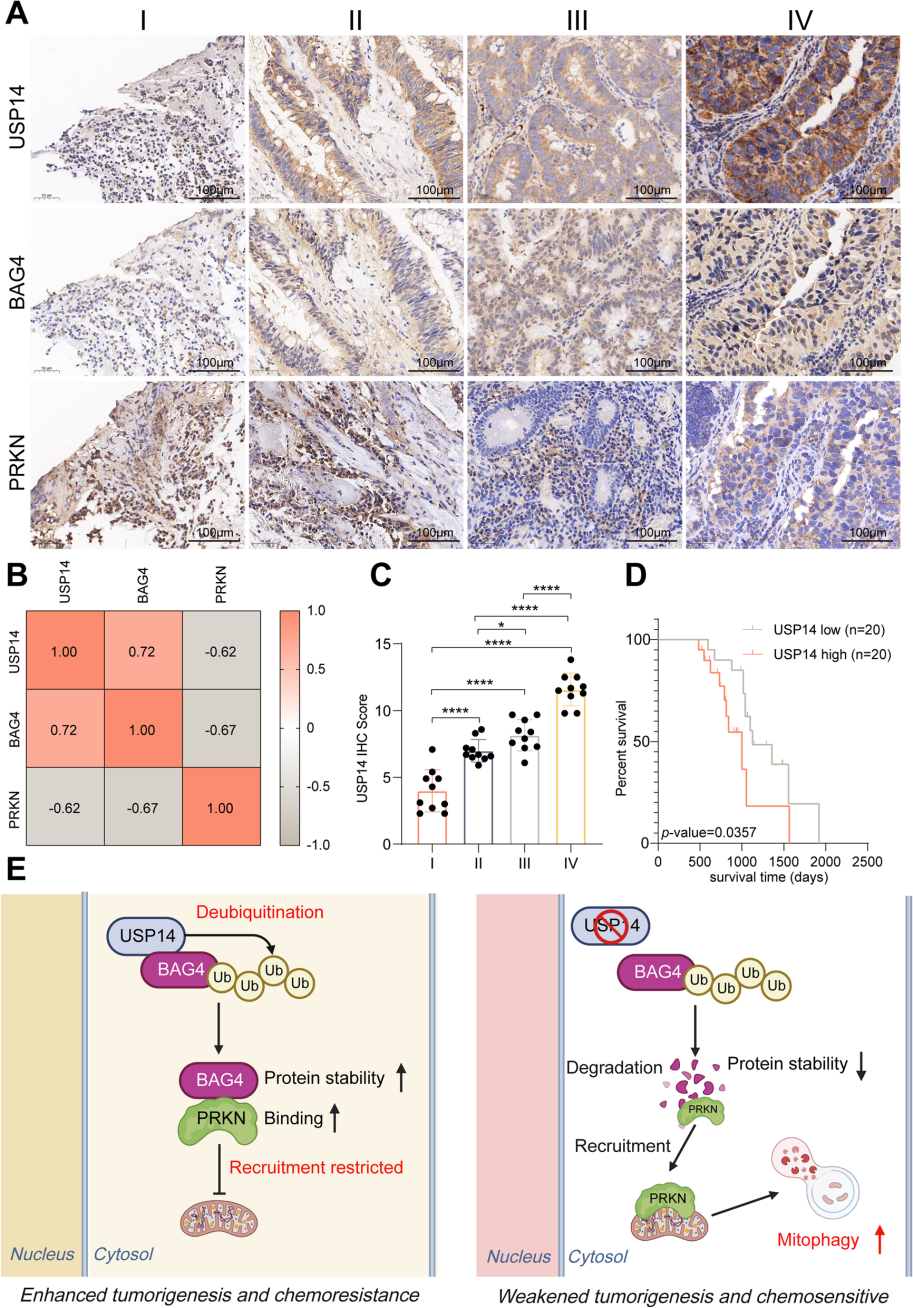
The Clinical Relevance of USP14, BAG4, and PRKN in CRC (MSI-H). Representative images of IHC staining of USP14, BAG4 and PRKN in clinical CRC (MSI-H) samples (**a**). Heat map shows the correlations of IHC data between USP14, BAG4 and PRKN expression (**b**). Quantitative analysis of USP14 expression in different stage of CRC (**c**). KM curve of USP14 high- and low-expression groups (**d**). Mechanism diagram (**e**). *P < 0.05, **P < 0.01, ***P < 0.001, ****P < 0.0001. Data are presented as the mean ± SD of three separate experiments

To elucidate the clinical relevance of the USP14/BAG4/PRKN regulatory axis in CRC (MSI-H), we first conducted an analysis of their expression profiles using RNA sequencing data from TCGA. The study found that USP14 and BAG4 were significantly overexpressed in tumor tissues compared to normal tissues, whereas PRKN expression was markedly reduced (Fig. S4a). To validate these findings at the protein level, we performed immunohistochemical (IHC) staining on tumor samples from 40 CRC (MSI-H) patients (Fig. 7a). The IHC results showed a strong positive correlation between USP14 and BAG4 expression, indicating that these proteins may be co-regulated in CRC (MSI-H) (Fig. 7b). In contrast, there was an inverse relationship between USP14/BAG4 and PRKN expression, supporting the hypothesis that BAG4 stability is modulated by USP14 in a manner that suppresses PRKN translocation. Further analysis across different tumor grades demonstrated a clear association between protein expression and disease severity: USP14 and BAG4 levels increased progressively with higher tumor grades, while PRKN expression decreased as the tumor grade advanced (Figs. 7c and S4b, c). This suggests a potential role of USP14 and BAG4 in promoting tumor progression, while PRKN may act as a tumor suppressor in CRC (MSI-H). From a prognostic standpoint, the expression levels of USP14 and BAG4 were significantly correlated with patient survival. High expression of USP14 and BAG4 was associated with poor overall survival, highlighting their potential as markers for aggressive tumor behavior (Figs. 7d and S4d). In contrast, patients with higher PRKN expression had a relatively better prognosis, although the *p*-value is not significant due to the limited sample size (Fig. S4e). The USP14/BAG4/PRKN axis is crucial in CRC (MSI-H) pathogenesis and may be a potential therapeutic target.

## Discussion

Post-translational modifications, particularly ubiquitination, play a crucial role in regulating protein activity and tumorigenesis by controlling protein degradation, stabilization, and signal transduction. This process is dynamically regulated by E3 ubiquitin ligases and deubiquitinating enzymes (DUBs), making them potential targets for cancer therapy. Mitophagy, a selective autophagy process, maintains cellular homeostasis by removing damaged mitochondria and has a dual role in cancer: either promoting tumor survival by reducing oxidative stress or suppressing tumorigenesis by preventing uncontrolled proliferation. GPR176 facilitates colorectal cancer progression through its interaction with GNAS, leading to the inhibition of mitophagy (Tang et al. 2023). The mitophagy protein PINK1 suppresses colon tumor growth through p53 activation (Yin et al. 2021). Aloe vera gel glucomannan induces colon cancer cell death via the PINK1/Parkin mitophagy pathway driven by mitochondrial damage (Zhang et al. 2022c). These insights underscore the therapeutic potential of targeting mitophagy in CRC treatment.

Mitophagy and ubiquitination are intricately linked cellular processes, with ubiquitination playing a crucial role in initiating and executing mitophagy (Jeon and Chung 2024). Proteins on the mitochondrial surface undergo ubiquitination, allowing their recruitment to autophagosomes, which subsequently fuse with lysosomes for mitochondrial degradation. Deubiquitinating enzymes (DUBs) play a regulatory role in mitophagy, demonstrated by USP8 and USP26 interacting with PINK1 and PRKN, and USP30 and USP33 inhibiting PRKN-mediated mitochondrial clearance (Sun et al. 2020; Wu et al. 2024; Okarmus et al. 2024; Niu et al. 2020). In this study, we first identified USP14 as the molecule most closely associated with mitophagy in CRC (MSI-H) through a DUB siRNA library screening. USP14, a member of the ubiquitin-specific proteases (USP) family, has been linked to the progression of various cancers, including colorectal, pancreatic, breast, and gastric cancers (Zhao et al. 2023; You et al. 2023). In CRC (MSI-H), USP14 deubiquitinates and stabilizes IDO1, preventing its degradation and promoting tryptophan metabolism, which leads to T-cell dysfunction (Shi et al. 2022). Additionally, USP14 enhances colorectal tumorigenesis by stabilizing JNK and regulating MAPK/JNK signaling (Du et al. 2023). It also interacts with fatty acid synthase (FASN), increasing its stability and influencing liver cancer cell proliferation, even in a FASN-independent manner (Chen et al. 2024). These studies highlight USP14’s key role across different cancer types, making it a potential target for cancer therapy. Through further analysis, we identified a direct interaction between USP14 and BAG4, where USP14 deubiquitinates and stabilizes BAG4 by specifically removing K48-linked ubiquitin chains from BAG4 at K403. BAG4 binds with PRKN, inhibiting PRKN recruitment into mitochondria and mitophagy (Fig. 7e). Additionally, USP14-mediated deubiquitination of BAG4 plays a significant role in tumorigenesis and chemosensitivity. Inhibition of protein synthesis prolonged BAG4’s half-life in USP14-deficient cells, and BAG4 upregulation reversed the effects of USP14 loss. The results identify USP14 as a potential therapeutic target in CRC (MSI-H).

In summary, our study highlights the role of USP14 in promoting tumor progression by regulating the BAG4/PRKN-mediated mitophagy pathway. These findings provide new insights into USP14’s involvement in mitophagy signaling in CRC (MSI-H). They emphasize the critical role of mitophagy in tumor development. Targeting USP14 through modulation of its activity or gene expression, and disrupting its interaction with BAG4 using interfering peptides, presents a promising therapeutic approach for CRC (MSI-H) treatment. Lastly, we acknowledge several limitations in this study. Firstly, the current investigation was conducted exclusively using MSI-High colorectal cancer cell lines (LoVo and SW48), which primarily represent the Consensus Molecular Subtype 1 (CMS1). Given the known molecular and clinical heterogeneity among CRC subtypes (CMS1-CMS4), our findings may not be directly generalizable to CMS2 (chromosomal instability), CMS3 (metabolic), or CMS4 (mesenchymal) subtypes. Thus, it remains uncertain whether USP14 inhibition would exhibit differential effects across various CRC subtypes. Future studies incorporating diverse CRC models representing other CMS subtypes, particularly CMS2 and CMS4, are necessary to comprehensively elucidate the role and therapeutic potential of USP14 across the broader CRC spectrum. Additionally, further mechanistic investigations in these different contexts may yield insights into subtype-specific responses to USP14-targeted therapies, thereby refining treatment strategies tailored to the molecular profile of CRC tumors.

## Conclusions

This study elucidates the pivotal role of USP14 as a regulator of mitophagy in CRC (MSI-H), contributing to tumorigenesis and chemoresistance. USP14 stabilizes BAG4 via K48-deubiquitination, hindering Parkin-mediated mitophagy, which results in the accumulation of damaged mitochondria and fosters tumor progression. High expression levels of USP14 are significantly associated with poor prognosis in CRC (MSI-H) patients. The study underscores the therapeutic promise of targeting the USP14/BAG4/PRKN axis to boost mitophagy and increase cancer cell sensitivity to chemotherapy, especially oxaliplatin. Overall, the study underscores the critical role of ubiquitination in modulating mitochondrial quality control and chemotherapy responses, offering new insights into the molecular mechanisms driving tumor pathogenesis and resistance. Targeting USP14 may represent a promising strategy for improving therapeutic outcomes in CRC (MSI-H).

## Supplementary Information

Additional file 1. Fig. S1 DUBs that exhibit differential expression between CRC (MSI-H) tissues and adjacent normal tissues from TCGA database. *P < 0.05, **P < 0.01, ***P < 0.001, ****P < 0.0001.

Additional file 2. Fig. S2 Ki67 and TUNEL staining reveal the role of USP14 in tumorigenesis and oxaliplatin sensitivity. The apoptotic cells without oxaliplatin treatment were measured by flow cytometry and analyzed by flow Jo (A and B). The protein expression levels of USP14 and BAG4 in the tumor tissue in Figure 2F (C). The Ki67 IHC staining reveals the impact of USP14 knockdown on tumor proliferative activity (D). Assessment of the impact of USP14 knockdown on oxaliplatin-induced apoptosis using TUNEL staining (E). The protein expression levels of USP14 and BAG4 in the tumor tissue in Figure 6F (F). The Ki67 IHC staining reveals the impact of BAG4 on USP14-mediated tumor proliferative activity (G). Assessment of the impact of BAG4 on USP14-mediated oxaliplatin-induced apoptosis using TUNEL staining (H). Quantitative analysis of (A) and (B) are shown in (I) and (J). Quantitative analysis of (D) and (E) are shown in (K). Quantitative analysis of (D) and (E) are shown in (L). *P < 0.05, **P < 0.01, ***P < 0.001, ****P < 0.0001. Data are presented as the mean ± SD of three separate experiments.

Additional file 3. Fig. S3 Validation of IU1 effect and protein-protein interaction assay comparing USP14 WT, USP14 inactive mutant (C114A) and BAG4.

Additional file 4. Fig. S4 The Clinical Relevance of USP14, BAG4, and PRKN in CRC (MSI-H). Relative expressions of USP14, BAG4 and PRKN between normal and tumor tissues in TCGA database (A). Quantitative analysis of BAG4 expression in different stage of CRC (MSI-H) (B). Quantitative analysis of PRKN expression in different stage of CRC (MSI-H) (C). KM curve of BAG4 high- and low-expression groups (D). KM curve of PRKN high- and low-expression groups (E). ns: not significant, *P < 0.05, **P < 0.01, ***P < 0.001, ****P < 0.0001. Data are presented as the mean ± SD of three separate experiments.

Additional file 5.

## Data Availability

No datasets were generated or analysed during the current study.
