# Supplementary figures and images for: USP14 inhibits mitophagy and promotes tumorigenesis and chemosensitivity through deubiquitinating BAG4 in microsatellite instability-high colorectal cancer
Source: Mol Med. 2025 May 2;31:163. doi: 10.1186/s10020-025-01182-w (PMC12048998; doi:10.1186/s10020-025-01182-w)

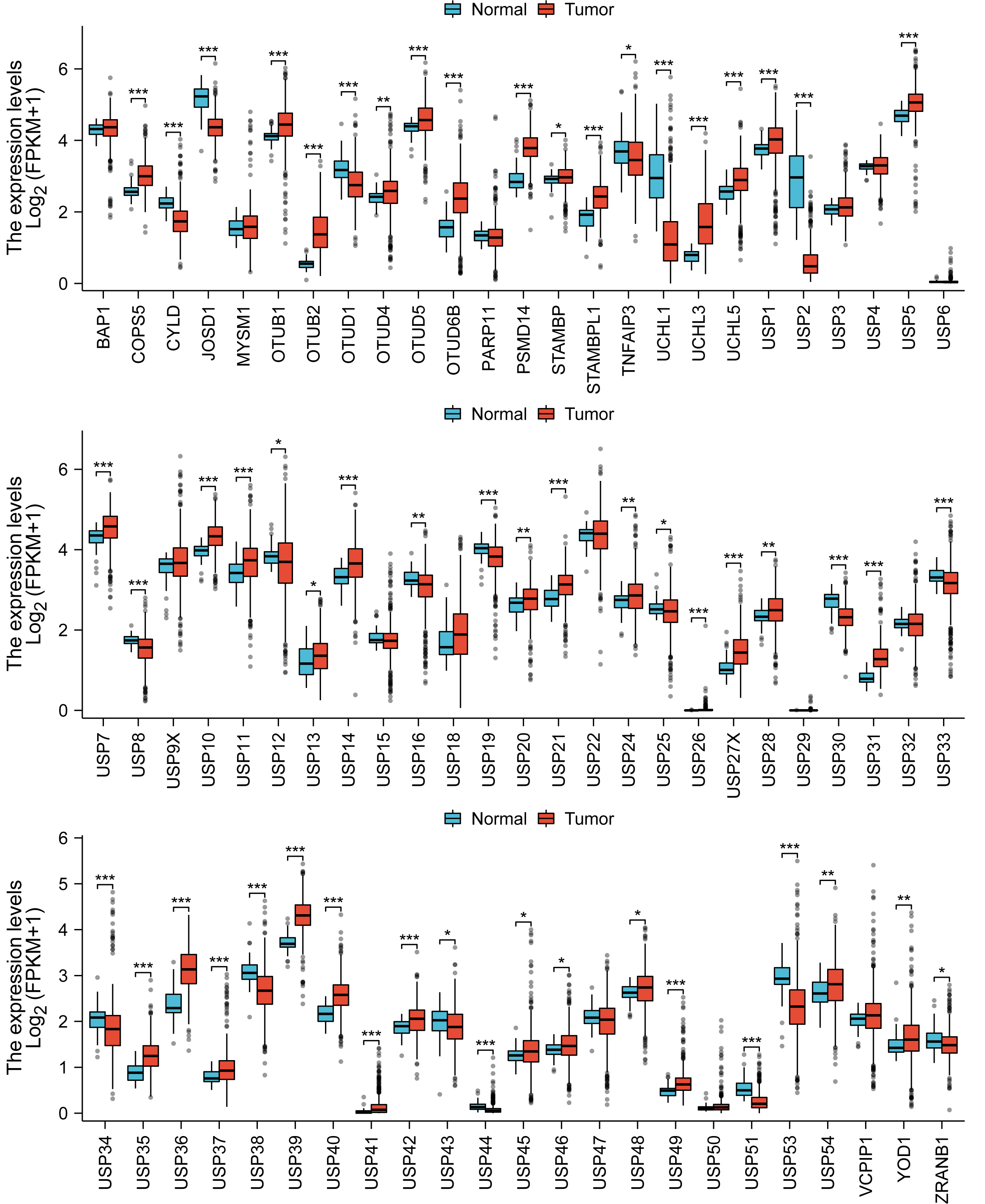

Supplement: Supplementary file 1 — Additional file 1. Fig. S1 DUBs that exhibit differential expression between CRC (MSI-H) tissues and adjacent normal tissues from TCGA database. *P < 0.05, **P < 0.01, ***P < 0.001, ****P < 0.0001. [file 10020_2025_1182_MOESM1_ESM.tif]

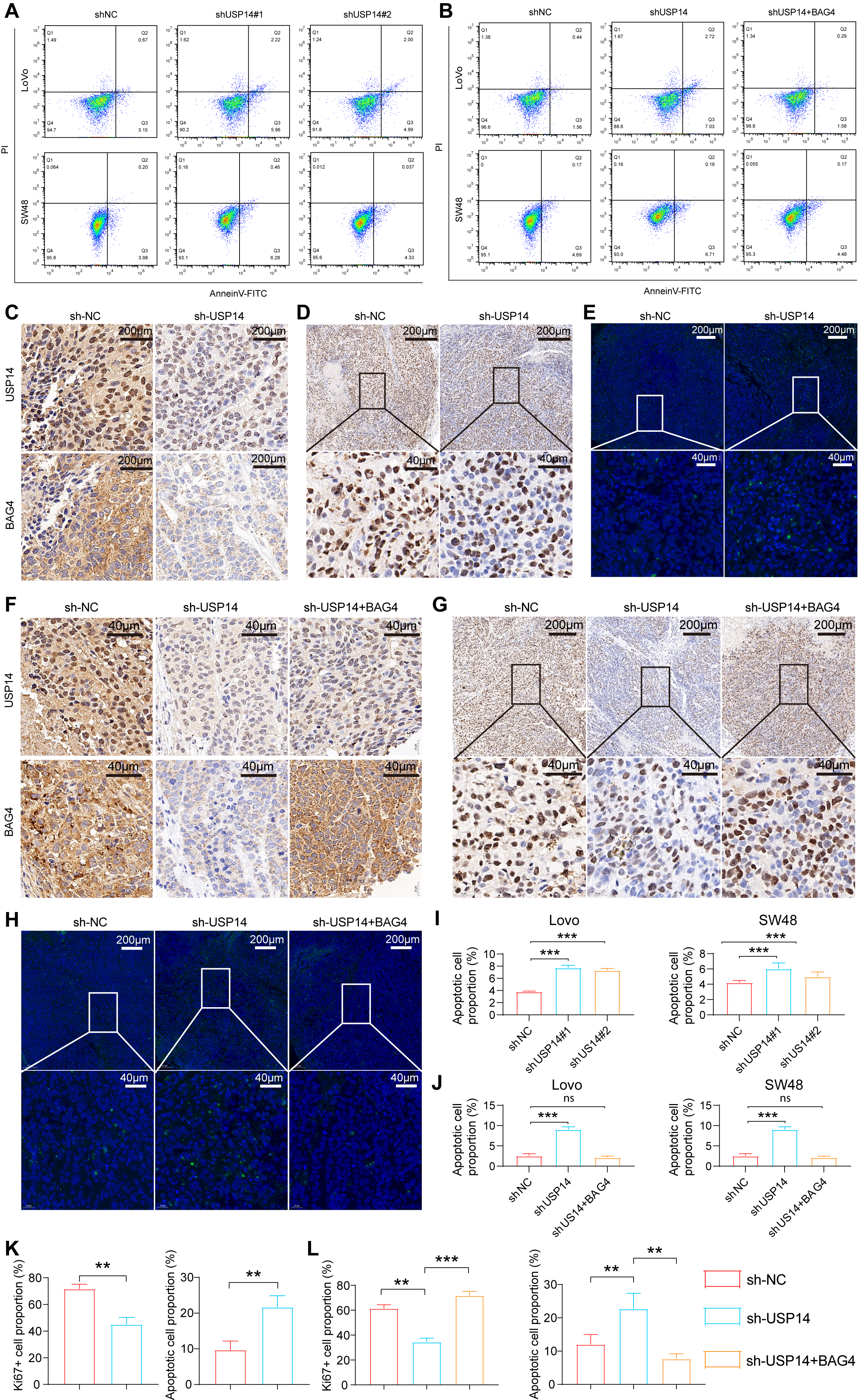

Supplement: Supplementary file 2 — Additional file 2. Fig. S2 Ki67 and TUNEL staining reveal the role of USP14 in tumorigenesis and oxaliplatin sensitivity. The apoptotic cells without oxaliplatin treatment were measured by flow cytometry and analyzed by flow Jo (A and B). The protein expression levels of USP14 and BAG4 in the tumor tissue in Figure 2F (C). The Ki67 IHC staining reveals the impact of USP14 knockdown on tumor proliferative activity (D). Assessment of the impact of USP14 knockdown on oxaliplatin-induced apoptosis using TUNEL staining (E). The protein expression levels of USP14 and BAG4 in the tumor tissue in Figure 6F (F). The Ki67 IHC staining reveals the impact of BAG4 on USP14-mediated tumor proliferative activity (G). Assessment of the impact of BAG4 on USP14-mediated oxaliplatin-induced apoptosis using TUNEL staining (H). Quantitative analysis of (A) and (B) are shown in (I) and (J). Quantitative analysis of (D) and (E) are shown in (K). Quantitative analysis of (D) and (E) are shown in (L). *P < 0.05, **P < 0.01, ***P < 0.001, ****P < 0.0001. Data are presented as the mean ± SD of three separate experiments. [file 10020_2025_1182_MOESM2_ESM.tif]

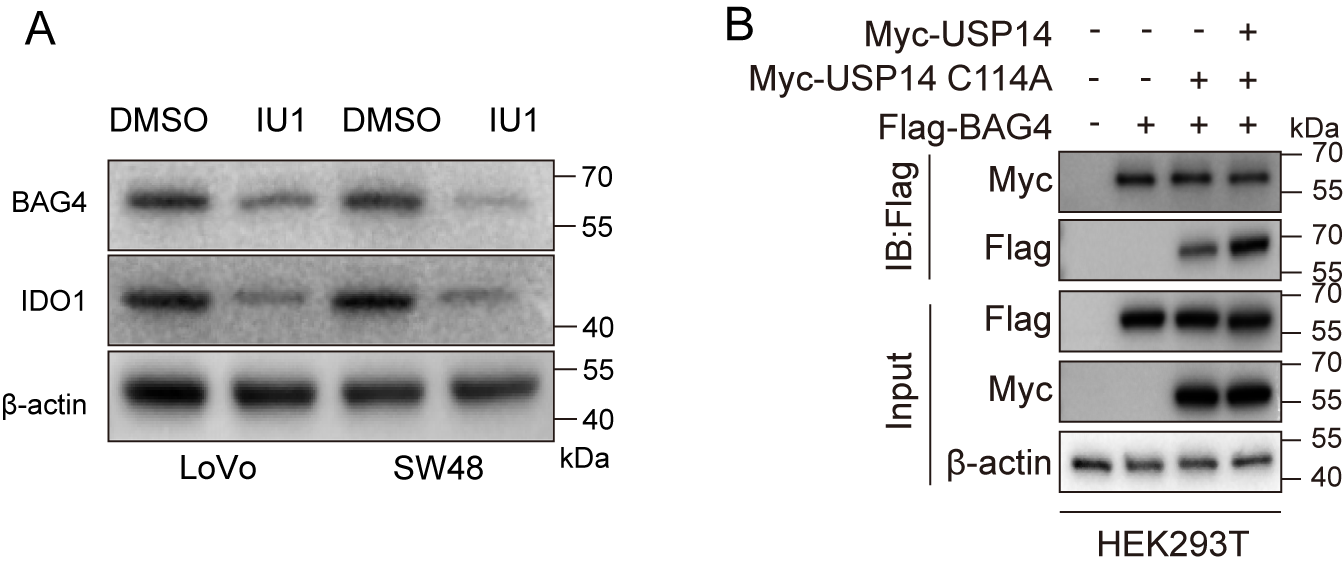

Supplement: Supplementary file 3 — Additional file 3. Fig. S3 Validation of IU1 effect and protein-protein interaction assay comparing USP14 WT, USP14 inactive mutant (C114A) and BAG4. [file 10020_2025_1182_MOESM3_ESM.tif]

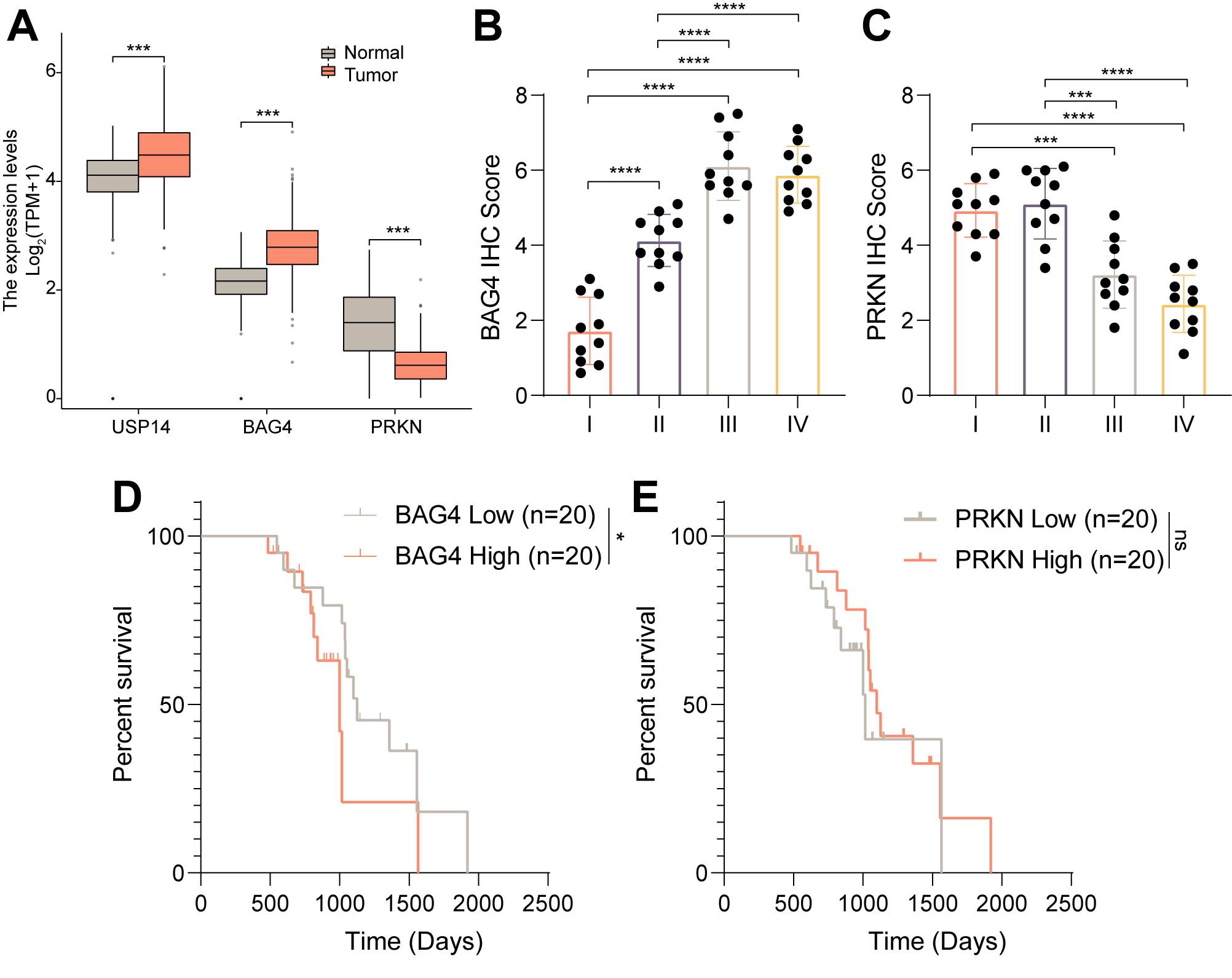

Supplement: Supplementary file 4 — Additional file 4. Fig. S4 The Clinical Relevance of USP14, BAG4, and PRKN in CRC (MSI-H). Relative expressions of USP14, BAG4 and PRKN between normal and tumor tissues in TCGA database (A). Quantitative analysis of BAG4 expression in different stage of CRC (MSI-H) (B). Quantitative analysis of PRKN expression in different stage of CRC (MSI-H) (C). KM curve of BAG4 high- and low-expression groups (D). KM curve of PRKN high- and low-expression groups (E). ns: not significant, *P < 0.05, **P < 0.01, ***P < 0.001, ****P < 0.0001. Data are presented as the mean ± SD of three separate experiments. [file 10020_2025_1182_MOESM4_ESM.tif]
